# Structural-functional lung imaging using a combined CT-EIT and a Discrete Cosine Transformation reconstruction method

**DOI:** 10.1038/srep25951

**Published:** 2016-05-16

**Authors:** Benjamin Schullcke, Bo Gong, Sabine Krueger-Ziolek, Manuchehr Soleimani, Ullrich Mueller-Lisse, Knut Moeller

**Affiliations:** 1 Institute of Technical Medicine, Furtwangen University, VS-Schwenningen, Germany; 2 Department of Radiology, University of Munich, Munich, Germany; 3 Engineering Tomography Lab (ETL) Department of Electronic and Electrical Engineering, University of Bath, Bath, UK

## Abstract

Lung EIT is a functional imaging method that utilizes electrical currents to reconstruct images of conductivity changes inside the thorax. This technique is radiation free and applicable at the bedside, but lacks of spatial resolution compared to morphological imaging methods such as X-ray computed tomography (CT). In this article we describe an approach for EIT image reconstruction using morphologic information obtained from other structural imaging modalities. This leads to recon- structed images of lung ventilation that can easily be superimposed with structural CT or MRI images, which facilitates image interpretation. The approach is based on a Discrete Cosine Transformation (DCT) of an image of the considered transversal thorax slice. The use of DCT enables reduction of the dimensionality of the reconstruction and ensures that only conductivity changes of the lungs are reconstructed and displayed. The DCT based approach is well suited to fuse morphological image information with functional lung imaging at low computational costs. Results on simulated data indicate that this approach preserves the morphological structures of the lungs and avoids blurring of the solution. Images from patient measurements reveal the capabilities of the method and demonstrate benefits in possible applications.

Electrical Impedance Tomography (EIT) is used to visualize the internal conductivity distribution of a domain from voltage measurements on the surface of the domain. In clinical context EIT is most often used to visualize regional ventilation of the lungs which has proven useful to reduce ventilator induced lung injury (VILI)^1^,^2^ and guide clinicians to set adequate PEEP levels for mechanically ventilated patients in the intensive care unit (ICU)^3^. In lung EIT an array of electrodes is attached around the patient’s chest. The electrodes are used to inject small alternating currents into the body and simultaneously measure the resulting voltages between pairs of electrodes. Usually the electrodes are attached equidistantly in a plane around the 5th intercostal space (ICS5), which seems to be less influenced by lung tissue shifts than more cranial or caudal positions for the electrodes^4^.

Compared to morphological imaging methods, such as X-ray computed tomography (CT) or magnetic resonance imaging (MRI), the spatial resolution of EIT is much lower (about 2–3 cm)^5^, whereas the temporal resolution is very high (up to 50 frames/s in commercially available devices). In comparison to established methods for functional imaging of the lungs, such as single photon emission computed tomography (SPECT) or positron emission tomography (PET), EIT is very cheap. Additionally it is non-invasive, radiation free, and currently the only technique capable for real-time and long-term bedside monitoring of regional lung ventilation^6^,^7^.

Besides the application in the ICU, EIT has recently been used in spontaneously breathing patients, suffering from asthma bronchiole^8^, cystic fibrosis (CF)^9^ or chronic obstructive pulmonary disease (COPD)^10^. It has been shown in these studies, that EIT is capable to identify functional abnormalities in obstructive lung diseases. However, in order to obtain a comprehensive insight into the pulmonary pathophysiology it is necessary to combine structural and functional data^11^, such as it is done in SPECT-CT or PET-CT.

Such combined imaging modalities simplify image interpretation; e.g. image fusion of PET and CT data substitutes error-prone cognitive allocation of anomalies and therefore improves diagnostics^12^. Additionally, it is common to use information obtained from one method to improve the reconstruction or the interpretability of the respectively other. E.g. the attenuation correction for PET imaging is derived from CT data^13^ and vice versa the detailed anatomic information from CT imaging is used to localize accumulation of the radiotracer^14^.

EIT has already been validated against CT^15^,^16^, SPECT^17^ and PET^18^, confirming that it is able to measure regional ventilation. Basic geometric relations between organs are preserved in EIT and images roughly correspond to the anatomy^19^.

The objective of this work is to fuse morphological and functional information to improve interpretability of EIT images. We propose a novel approach to include detailed prior information about the contour of the thorax as well as the shape of the lungs in the image reconstruction process. This can be regarded as ‘patient specific EIT imaging’, where resulting images can easily be superimposed with CT data and thus improve readability for clinicians. Morphological changes can be directly correlated with changes in ventilation distribution or other functional images derived from EIT. It is demonstrated in simulations and measurements on patients how advantages of both imaging modalities (CT and EIT) can be combined to improve the potential utility of functional lung image in clinical applications.

## Methods

### Classical approach for image reconstruction

The EIT reconstruction problem is ill-posed, arbitrarily small perturbation in the boundary voltages may create arbitrarily large perturbations of the solution, i.e. the conductivity distribution^20^. Therefore, to obtain useful estimates of conductivity distribution a regularization scheme is needed^21^. Additionally the EIT problem is non-linear, which means that boundary voltages are non-linearly related to internal conductivities^22^.

Several reconstruction methods for EIT imaging have been developed, all showing one or the other advantage. Most of them have been developed for special purposes, e.g. absolute EIT imaging^23^, compensation of motion artifacts^24^, compensation for faulty electrodes^25^, 3D imaging^26^, methods considering temporal correlation to reduce image noise^27^ or utilizing Kalman filter approaches to track fast changes in conductivity distribution^28^. Other algorithms are based on expert defined figures of merit^29^ or on eigenimages from CT data as training sets^30^. Blurring of reconstructed images is prevented in approaches using the level set method^31^ or Total Variation Regularization^32^. Constantly increasing computational power and parallel computing allow fast reconstruction of nonlinear EIT^33^ and direct methods, such as the d-bar algorithm^34^.

The below described one-step Gauss-Newton method represents a broad group of structurally similar algorithms for image reconstruction, that have been widely applied in EIT^29^,^35^. We therefore use this ‘classical’ approach for both: a) to demonstrate the benefits of the overall approach of direct structure integration into EIT reconstruction and b) to relate the outcome to generally accepted standard EIT modalities.

A common method to estimate the conductivity from voltage measurements on the boundary is to discretize the domain into a finite element model (FEM) of *n*_*elem*_ piecewise constant regions. Rather constructing absolute values of conductivity most of the clinical EIT research uses time-difference EIT, where the change in conductivity distribution **x** = ***σ*** − ***σ***^*baseline*^ between two times is calculated from voltage changes **y** = **v** − **v**^*baseline*^. The vectors ***σ***^*baseline*^ and **v**^*baseline*^ denote the conductivity and boundary voltage at an appropriately chosen baseline; vectors ***σ*** and **v** contain the conductivity and voltages at another time. In practice it is common to set the baseline at functional residual capacity (FRC) or for mechanically ventilated patients at positive end-expiratory pressure (PEEP). Since this conductivity distribution is usually not known it is common to set ***σ***^*baseline*^ = **1**, which may lead to significant errors. It is recommended to consider the conductivity distribution obtained e.g. from CT or MRI images to improve image quality^36^. In normalized time-difference EIT the difference in measured voltage **y** is normalized, such that 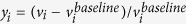, where the index *i* denotes the i-th element of the vectors, respectively. In the remainder of this paper we use normalized values for voltage change.

The classical approach for reconstruction of conductivity distribution in difference EIT is


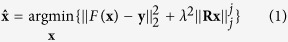


where 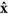 is the reconstructed conductivity change. The non-linear forward model *F*(**x**) maps changes in conductivity distribution to voltage changes at the electrodes. For small changes in conductivity the change in boundary voltage can be approximated by a linear mapping such that 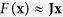 which leads to


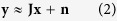


where **n** is the noise of the measurement system and **J** is the Jacobian. Each element of **J** relates a small voltage change at the i-th position on the measurement vector **y** to a conductivity change of the j-th FEM element and is calculated as:


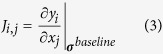


The Jacobian **J** is depending on the linearization point ***σ***^*baseline*^. To avoid errors caused by an assumed homogeneous distribution ***σ***^*baseline*^ we considered different conductivities for lung tissue and remaining tissue, throughout this paper. This is described in more detail below. The Jacobian **J** does as well depend on the current stimulation pattern. In this paper we use the adjacent current stimulation pattern, which applies current and measures voltages between neighboring pairs of electrodes. For *n*_*elec*_ electrodes this results in *n*_*meas*_ = *n*_*elec*_ · (*n*_*elec*_ − 3) voltage measurements (of which 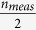 are independent) for every frame and thus 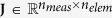.

Thus, the linearized problem is formulated as:


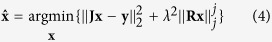


The first term is often called the ‘data fidelity term’, the second term is called ‘regularization term’ and adds, depending on the choice of **R**, penalties to reconstructed conductivity distributions. The level of regularization is controlled with the scalar value *λ*. The norm of the regularization term is specified with *j*. Mathematically the ‘regularization term’ is used to replace the ill-posed problem by a nearby well-posed^37^.

There are numerous publications about the choice of **R**, which can be regarded as a way of implementing prior knowledge into the solution. Most of these approaches penalize the solution such that a given structure of the solution is obtained. This leads to small, smooth or slowly changing solutions 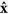
38. In this paper we either use the Tikhonov prior **R**_*Tik*_ = **I**, which leads to solutions with smaller norms, or the Laplace prior **R**_*LP*_^39^, to penalizes non-smooth solutions.

If the *l*_2_-norm is used for the regularization term of equation (4) the estimated change in conductivity 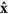 can be calculated in a closed form


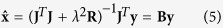


Matrix **B** is called ‘reconstruction matrix’ which maps voltage changes **y** to changes in conductivity distribution 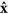. In real-time systems **B** is usually pre-calculated, e.g. during startup of the device. The reconstruction of 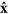 is thus realized as a trivial matrix multiplication, which is one of the reasons for real-time capability of EIT.

### Introduction to Discrete Cosine Transformation (DCT)

The Discrete Cosine Transformation (DCT) is an established method in image processing. A popular application is the JPEG image compression, where images are converted to a frequency-domain representation. The idea of DCT is to represent a signal as a weighted sum of basis functions, whereas the basis functions are cosine functions with different frequencies *p* and *q* in x-direction and y-direction, respectively. For a two dimensional image **A** with *M* rows and *N* columns the DCT is defined as follows:


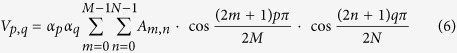


where


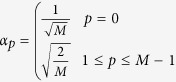


and


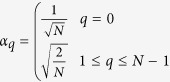


The resulting matrix **V** contains the DCT coefficients. A lossy compression of the image **A** can be reconstructed using only a subset of the DCT coefficients.


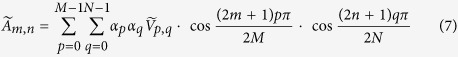


where 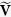 is a matrix of the same size as **V** but contains only few nonzero elements (e.g. the k-largest elements of **V**).

**Figure 1:**
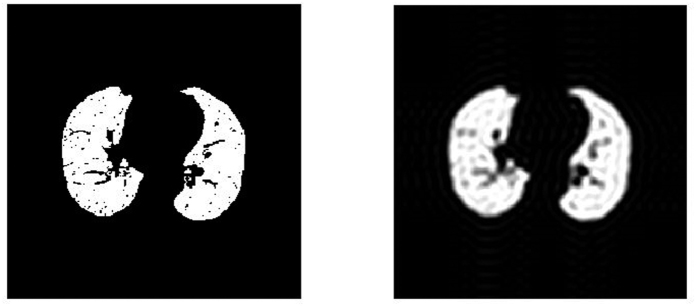
Left: Original image with 256 × 256 pixels. Right: Reconstruction using only the 50 lowest frequency components.

Figure 1 is an example of a DCT with a relatively high compression ratio of 96%. Figure 1 (left) shows the original image (256 × 256 pixels), Fig. 1 (right) is a reconstruction with the 50 lowest frequency components in each direction, which means that 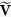 contains zero values for row and column indices greater than 50.

### Dimensionality reduction using DCT and implementation of prior knowledge

The idea of this paper is to represent the solution of the inverse problem in terms of cosine basis functions. Only a subset of DCT coefficients is considered for reconstruction, which reduces the dimensionality of the problem. We only need to solve for the coefficients of cosine basis functions instead of all elements in the FEM mesh. This reduction of dimensionality is achieved by multiplying the Jacobian 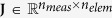 by a matrix 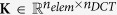, where *n*_*DCT*_ is the number of DCT coefficients used for reconstruction and *n*_*DCT*_ ≪ *n*_*elem*_. In this paper we use the same number of frequencies for the x-direction *n*_*X_DCT*_ and y-direction *n*_*Y_DCT*_ such that 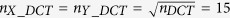, which means that only *n*_*DCT*_ = 225 DCT coefficients are reconstructed. This is substantially less than the number of elements in typical FEM meshes.

The above formulation of the DCT is expressed as:


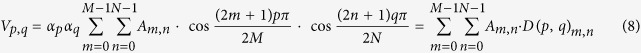


with **D**(*p, q*) being the cosine function at frequencies *p* and *q*. To introduce prior knowledge about the shape of the lungs, and thus the possible conductivity distribution we generate the matrices **C**(*p, q*), which are


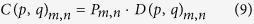


with 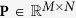 being a binary image of the lungs with


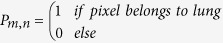


The binary image of the lungs can be obtained e.g. by separating the lungs from CT data. If no CT data is available it might be suitable to define lung-shaped regions of possible conductivity change. Each column **k**_*j*_ of **K** is created as follows


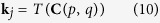


where 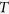 is the map assigning each pixel in the matrix 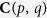 to the finite element which covers the corresponding pixel. The values of the index 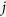 depend on 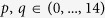 as follows:


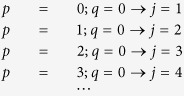



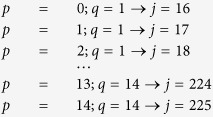


Please note that the number of FEM elements representing the lung tissue might be smaller than the number of pixels categorized as lung tissue in the matrix **C**(*p, q*). Although this means that not all elements of **C**(*p, q*) are used, the columns of **K** are still linearly independent, which means that **K** is a valid basis of the selected low frequency subspace of the DCT representation.

**Figure 2:**
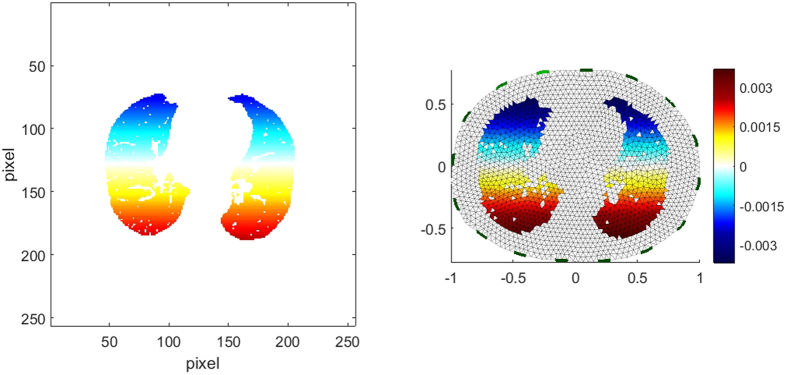
Left: Representation of the matrix C with *p* = 0 and *q* = 1. Right: Visualization of the corresponding column **k**_16_ of **K** with the FEM model of the thorax.

For better understanding the exemplarily illustration of the result of the element-wise multiplication of a cosine function and a binary lung image according to equation (9) is depicted in Fig. 2 (left), according to equation (10) the matrix **C**(*p, q*) is mapped to the elements of the FEM model, which is represented by a column of matrix **K** (Fig. 2 (right)).

Each column **j**_*j*_ of the Jacobian **J** describes how change in conductivity of the j-th FEM element influences the measured changes in boundary voltages. Hence the image reconstruction algorithm calculates a linear combination of the columns of **J** that best fits the measured voltage change **y**. Generating a new Jacobian **J**_*DCT*_ = **J** · **K**, where 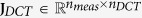 leads to a different interpretation of the columns. In this case each column **j**_*DCT, j*_ describes how a certain distribution of conductivity change **k**_*j*_ influences the measured changes in boundary voltage. This results in a new formulation of equation (5) such that:


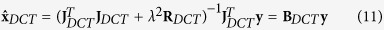


where 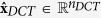 can be interpreted as reconstruction of DCT coefficients. In other words the j-th element of the vector 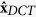 explains to what extend the relative voltage change **y** is induced by the j-th column of **K**. Accordingly, the reconstruction matrix **B**_*DCT*_ maps voltage changes **y** to DCT coefficients.

Prior knowledge about the DCT coefficients can be incorporated in **R**_*DCT*_. By setting 
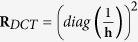
, the solution of equation (11) is drawn towards an expected solution with **h** being the DCT coefficients of the expected solution, which might be obtained from CT or MRI data. Throughout this paper we set **R**_*DCT*_ = **I**, which is the identity matrix and thus the classical approach for Tikhonov regularization^40^.

The result 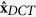 can now be used to reconstruct an image 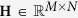 with the same resolution as the lung shape obtained from the original CT image. Therefore the matrices **C**(*p, q*) have to be multiplied with the corresponding values of 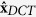 and summed element by element:


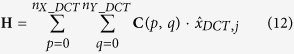


where the subscript *j* of 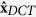 corresponds to the values of *p* and *q* as already described below equation (10).

The image **H** contains information about the lung shape. It is therefore a method to incorporate information of ventilation distribution obtained from boundary voltage measurements of an EIT device with patient specific physiological information about the lung shape. The cognitive allocation of ventilation distribution to lung areas in CT images can be replaced with the described approach and resulting images can directly be superimposed with CT images.

In order to be able to compare different approaches for image reconstruction we used the noise figure (NF) calculation^41^ to find values for the hyperparameter *λ*.


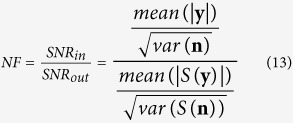


where the function *S* is used to map changes in voltage **y** or noise **n** to conductivity change. Depending on the approach used for reconstruction *S* is realized either as described in equation (4) or as in equation (11) and (12) if images are reconstructed with the DCT approach. A bisection search technique was used to find the corresponding values for *λ* to obtain a noise figure of NF = 0.5 for all reconstructions in this article.

### Data generation

**Figure 3:**
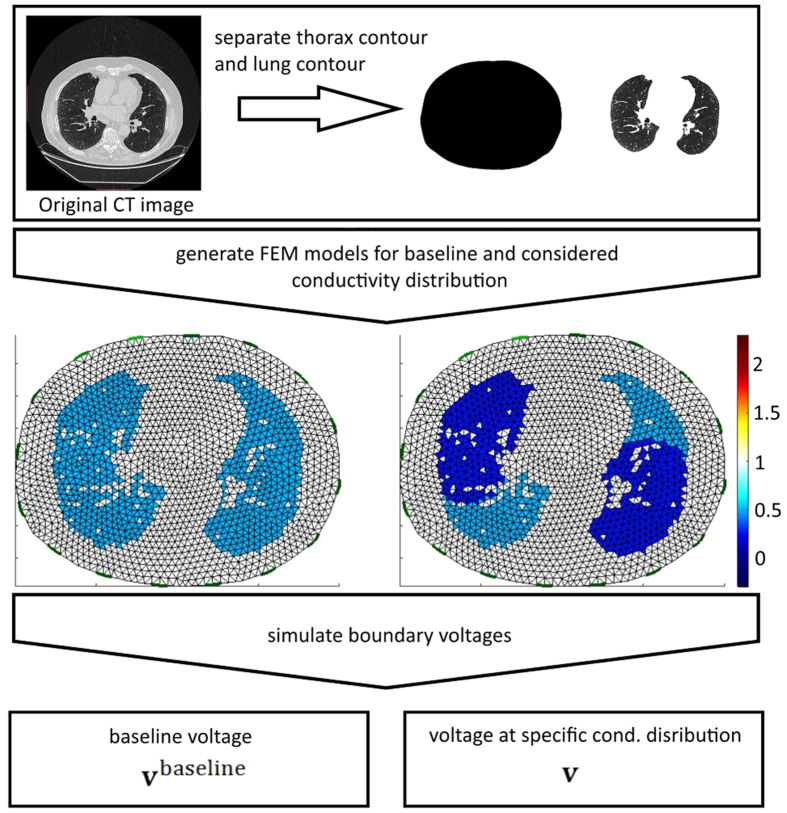
Procedure of voltage simulation for different configurations of the lungs: Thorax shape and lung shape were derived from CT data and used to create FEM models to simulate boundary voltages. Different ventilation patterns were simulated by setting selected FEM elements to *σ* = 0.5 for non-ventilated lung-tissue and *σ* = 0.25 for ventilated lung-tissue.

Data of the boundary voltage were simulated using MATLAB R2015a (Mathworks, Natick, MA) and the EIDORS toolbox^42^. FEM meshes were generated with NETGEN^43^. To test the above described approach on artificial data we used a CT dataset of a subject (male, 68 years) to simulate boundary voltages. We used the adjacent current stimulation pattern because the used hardware in the subsequent part of this article also runs with the adjacent current stimulation pattern, although this method for current injection and voltage measurement is not optimal^44^. The thorax shape at 5^th^ intercostal space was derived from CT data as well as the shape of the lungs. Subsequently the thorax shape was used to generate a FEM mesh with 3570 elements. The conductivity of the elements belonging to the lungs was set to *σ* = 0.5, remaining elements were set to *σ* = 1. The corresponding voltage **v**^*baseline*^ of this conductivity distribution was simulated. Different distributions of ventilation were simulated by changing the conductivity of selected elements to *σ* = 0.25. Again boundary voltages **v** were simulated, leading to the normalized change in voltage **y**. The procedure of generating different ventilation patterns is depicted in Fig. 3.

**Figure 4:**
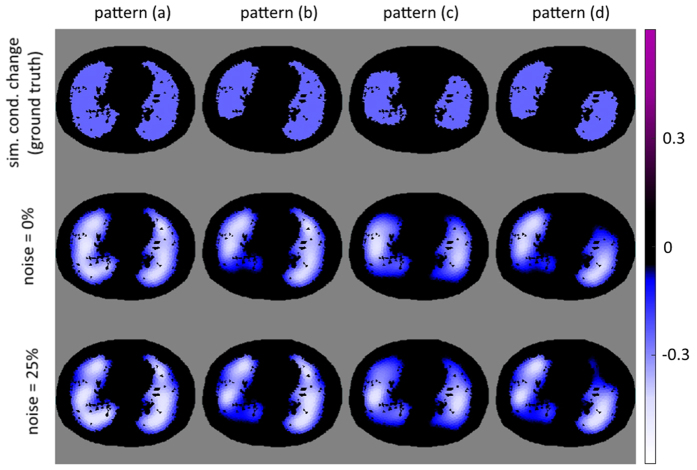
Upper row: Patterns of conductivity change used for simulation of boundary voltages (ground truth). Middle row: Reconstruction of the conductivity change from simulated voltages. Lower row: Reconstructed conductivity changes with 25% Gaussian noise on the simulated voltages.

Four different ventilation patterns were created: a) homogeneous ventilation of complete lung, b) no ventilation in dorsal right lung, c) no ventilation in the most ventral and most dorsal parts of both lungs, and d) no ventilation in ventral left lung and dorsal right lung. Pattern a) is used to evaluate the behavior of the algorithm on lung-healthy subjects whereas b) describes a typical pattern e.g. of patients under general anesthesia when the dorsal parts of the lungs collapse and lead to atelectasis. Pattern c) represents the simultaneous occurrence of alveolar hyperinflation in the most ventral parts and atelectasis in the most dorsal parts of the lung which may occur at the same time in critically-ill patients^45^. Pattern d) is rarely seen in real patients and is used to demonstrate the capability of the described algorithm. Figure 4 (upper) shows all used ventilation patterns.

Besides the four ventilation patterns different states of atelectasis of the dorsal lungs were simulated. Overall 25 datasets were generated, starting at 0% until 50% of the lungs in anterior-posterior direction were considered as collapsed. Elements belonging to collapsed lung regions were assigned to *σ* = 0.5, ventilated elements to *σ* = 0.25.

Another FEM mesh was used for the reconstruction of conductivity change to avoid the ‘inverse crime’^46^.

### Patient data

In order to evaluate the performance of the described method we also used real measured retrospective EIT data covering a clinical context. EIT data were recorded for different clinical studies on 5 patients (male, 66y ± 3) with different types of diseases. Informed consent was obtained from all subjects, the methods were carried out in accordance with the approved guidelines and regulations. The studies were approved by the local ethics committee of Kiel University and LMU Munich. The patients were either spontaneously breathing or connected to a mechanical ventilator while a PEEP step was performed. The Goe-MF II device (Viasys Healthcare, Höchberg, Germany) or the PulmoVista500 (Dräger Medical, Lübeck, Germany) were used to measure the boundary voltages. Data were collected at ICS5. For all patients an actual CT dataset was available which was used to determine the contour of the thorax and the shape of the lungs at the position of the electrodes. Table 1 provides an overview of the patient’s diseases and the used EIT device.

### Evaluation of image quality

For the simulated ventilation patterns the pixel values of the considered ventilation patterns and the pixel values of the reconstructed images were compared. Since we are most interested in conductivity decrease we define that pixels *H*_*m, n*_ in the reconstructed image **H** with a lower value than a heuristically defined threshold are classified as ‘decrease in conductivity’ (pixel set to 1). Other pixels are classified as ‘no change in conductivity’ (pixel set to 0). The threshold is defined with a parameter *α* as described below:


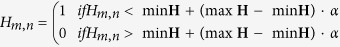


With this classification we are able to distinguish the fraction of pixels which correctly depict the underlying conductivity change and quantify the amount of correctly and incorrectly reconstructed data. The pixels are clustered in four groups, where G1 and G4 correspond to correct conductivity reconstruction; G2 and G3 correspond to incorrect reconstruction.

- G1 = Pixel correctly depicts decrease in conductivity
- G2 = Pixel wrongly depicts decrease in conductivity
- G3 = Pixel wrongly depicts no change in conductivity
- G4 = Pixel correctly depicts no change in conductivity

## Results

### DCT image reconstruction for different ventilation patterns

The performance of the DCT-approach with four different patterns of ventilation is depicted in Fig. 4. The upper row depicts the conductivity change used in the simulation of boundary voltages (ground truth). No change in conductivity is shown in black. Tissue with a change in conductivity from *σ* = 0.5 to *σ* = 0.25, as described above, is shown in light blue. The middle row and lower row represent reconstructions using the DCT approach without noise and with 25% Gaussian noise added, respectively.

**Figure 5:**
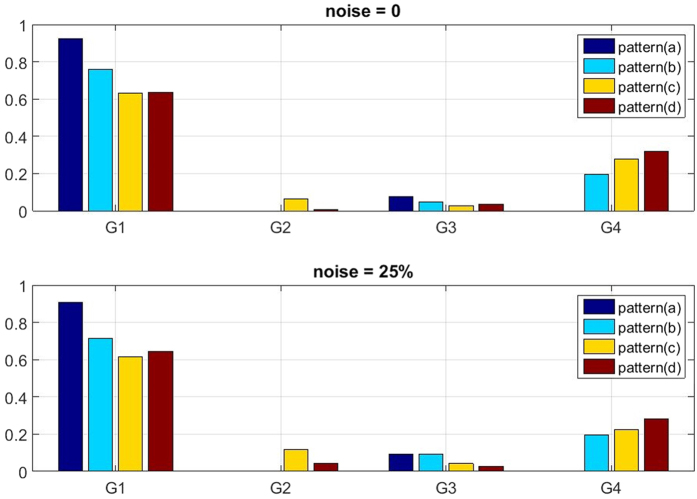
Fraction of pixels that are correctly classified as conductivity change (G1) or no change in conductivity (G4). Classes G2 and G3 respectively show the amount of pixels wrongly depicting changes in conductivity although there is none and pixels that show no changes in conductivity although the conductivity has changed. Threshold parameter *α* = 0.65. Upper graph is calculated with no noise, in the lower graph 25% Gaussian noise is added.

The fraction of correctly (clusters G1 and G4) or wrongly (clusters G2 and G3) classified pixels is visualized in Fig. 5. Threshold parameter was set to *α* = 0.65. The fraction of correctly classified pixels for the four considered patterns without noise is: (a) 0.91; (b) 0.99; (c) 0.91; (d) 0.96. With 25% of artificial noise these values change to: (a) 0.90; (b) 0.96; (c) 0.91; (d) 0.96.

### Simulation of atelectasis

**Figure 6:**
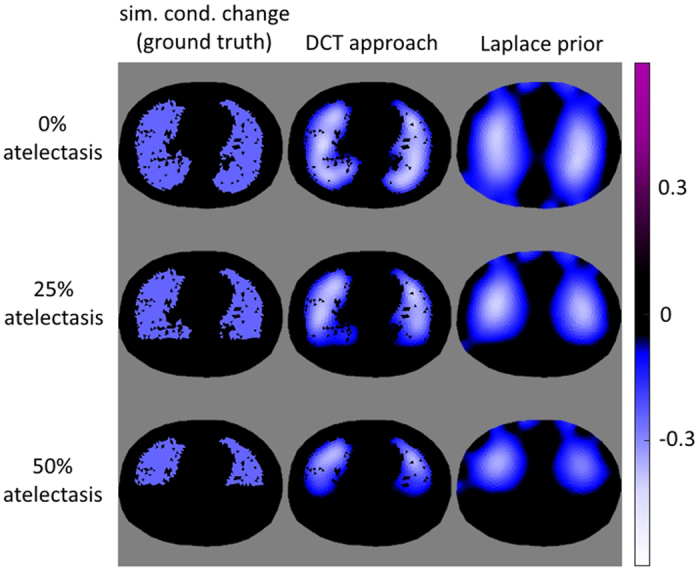
Left column: Atelectasis patterns used for voltage simulations (ground truth conductivity change). Middle column: Reconstruction of conductivity changes using the DCT-approach. Right column: Reconstruction of conductivity change using the classical approach and Laplace prior.

Figure 6 shows the difference between the DCT-approach for image reconstruction compared with the classical solution using the Laplace prior for three ventilation patterns, representing 0%, 25% and 50% of atelectasis of the dorsal lungs.

The Laplace prior in Fig. 6 (right) smoothes the reconstructed values of conductivity change, which causes a loss of morphological structures of the lungs. In contrast the DCT-approach in Fig. 6 (middle) utilizes the morphological information form CT data and reconstructs the conductivity change onto these structures.

**Figure 7:**
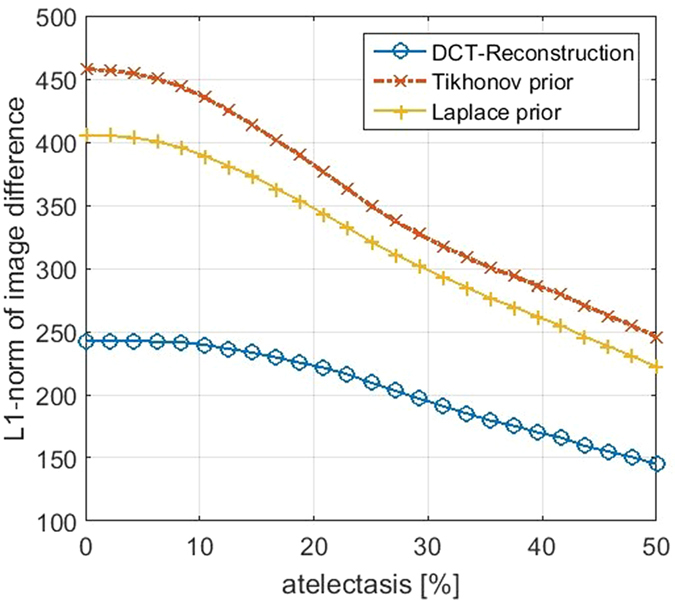
*l*_1_-norm of difference of reconstructed images and true conductivity distribution for different amounts of simulated atelectasis. Blue circle: DCT reconstruction, red x-marker: classical approach with Tikhonov prior, yellow plus-marker: classical approach with Laplace prior.

Computing the *l*_1_-norm of the difference of reconstructed image to the ground truth data confirms this observation, as depicted in Fig. 7. Using the DCT reconstruction results in a reduced *l*_1_-norm of image difference compared to the classical approach using Tikhonov prior or Laplace prior.

### Patient data

**Figure 8:**
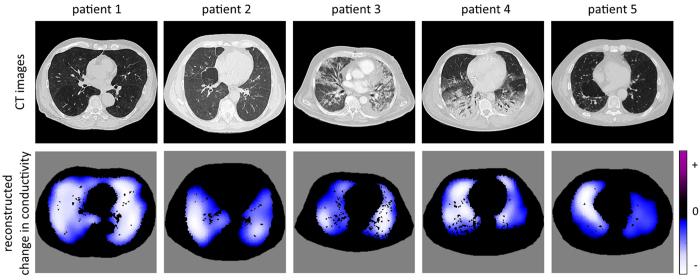
Upper row: Lung and thorax shape of the patients used for reconstruction. Lower row: Reconstructed images of conductivity change based on the DCT approach.

Reconstructed images of conductivity change for the 5 subjects are shown in Fig. 8. The pixels belonging to the lung were extracted from CT values as well as the thorax shape of the considered slice.

**Figure 9:**
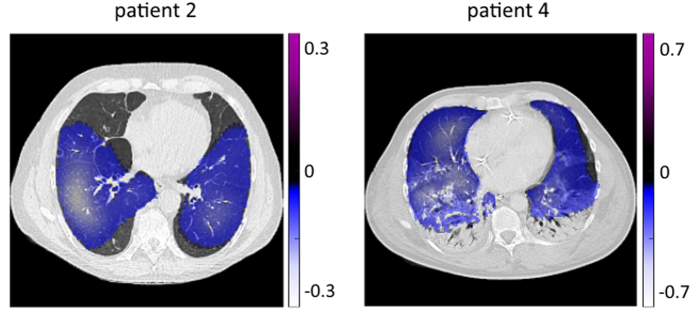
Superposition of changes in conductivity distribution obtained by the described DCT approach and CT images in a patient with emphysema in the ventral lungs (left) and atelectasis in dorsal lungs (right).

The images use the same color scheme as in the simulations above, where white color depicts large negative changes in conductivity, light blue color depicts small negative changes in conductivity. Positive changes in conductivity are depicted in purple, but are rarely seen during inspiration. For the purpose of illustration we do not depict the color scale in Fig. 8, which is different for every patient (Fig. 9).

Although we do not know the correct distribution of conductivity change, the results of Fig. 8 seem to be reasonable. Patient 1 did not show any abnormalities in the lung CT; hence we expect to obtain homogeneous ventilation distribution. The reconstructed data confirm this. Patient 2 shows areas of pulmonary emphysema in both ventral lungs. We therefore expect lower values of ventilation, which is confirmed by the EIT data. Patient 3 has atelectasis in the dorsal left lung regions determined from lung CT. This can as well be seen on the reconstructed EIT data. Also Patient 4 has atelectasis, which can be recognized in both lungs from CT images and EIT reconstructions. Patient 5 has pulmonary emphysema in the dorsal right lung while the left lung is hyperinflated. Ventilation distribution obtained with EIT confirms lower ventilation of the left lung and reveals that the upper left lobe shows almost no ventilation.

A superposition of conductivity change reconstructed with the proposed DCT algorithm and a CT image is depicted in Fig. 9 for patients 2 and 4. It can be seen in Fig. 9 (left) that both ventral lungs show no change in conductivity. Figure 9 (right) reveals that the most dorsal regions of both lungs show no change in conductivity, which strengthens the diagnosis of atelectasis.

## Discussion

In this paper we present a method to incorporate patient specific structural information (e.g. CT data) in the reconstruction process of EIT imaging. It is demonstrated in simulations and as well in real patient data that the morphological structure of the lungs is preserved in the reconstructions. Blurring of the solution into non-lung regions is prevented and resulting images can easily be superimposed with high-resolution morphological data, as it is done already in combined imaging methods, such as SPECT-CT or PET-CT. This facilitates a comprehensive insight into the pathophysiology of lungs, since regional information about the functional status can directly be correlated with the morphology of the lungs. Furthermore, the use of DCT reduces the dimensionality of the problem, which is computationally beneficial for large scale problems (e.g. dense mesh 3D EIT) or iterative reconstruction methods^47^.

Other approaches have been proposed earlier that use detailed and patient specific anatomical information in the image reconstruction. As demonstrated on simulation models it is possible to enhance the sharpness of the images using structural prior information, incorporating anisotropic smoothness constraints in the regularization^48^ or Gaussian anisotropic filters^49^, relaxing the smoothness in direction of expected change. Compared to our approach there is no explicit reduction of the dimensionality of the problem. Other approaches using a small number of simple fixed conductivity sets to model the physiology of the considered domain, to reduce dimensionality have been described^50^,^51^. These studies use the shape of the lungs and the contour of the thorax, derived from CT or MRI data, to group FEM elements and assigning them the same electrical properties. The results of these methods for reduction of dimensionality are strongly biased^52^ and, contrary to our approach, inhomogeneities of the lungs cannot be visualized. In other articles^21^,^53^ nine different states of the lungs and the heart are used to generate orthonormal basis functions. However, in these methods the underlying truncated basis for the reconstructed conductivity is not capable to visualize more complex distributions of lung ventilation. The feasibility of reduced-order models using orthogonal decomposition of conductivity and potential distributions has been demonstrated in simulations and tank measurements^47^, but no individual structural information is embedded in the reconstruction. An approach that shows the capabilities of image fusion of CT and EIT data utilizing a coregistration algorithm is described for measurements on a phantom^54^. However, neither structural information is directly used in the EIT reconstruction nor is the dimensionality of the problem reduced.

The stability and low computational costs of linear reconstruction algorithms are reasons for their important role in clinical applications^30^. The presented DCT approach is based on the simple, but probably most often used^35^ reconstruction method, which is the one-step Gauss-Newton solver. Thus it is not directly applicable to all of the above mentioned algorithms, e.g. the d-bar method^34^ or Total Variation Regularization^32^. However, we are confident that the DCT approach can be combined with some of the existing linear methods. As an example temporal one-step solvers^27^ could be adapted to reconstruct DCT coefficients instead of conductivity changes for every FEM element. In a similar way Kalman filter approaches^28^ could be extended to benefit from the advantages of the proposed approach. Furthermore, the DCT approach could easily be extended to 3D-EIT, where the reduction of dimensionality might facilitate real-time reconstruction with sufficient resolution.

Its radiation free operation is often mentioned as an advantage of EIT compared to other imaging techniques. However, in combination with structural imaging described in this paper we utilize morphological information of the lungs, which is usually acquired with CT. The described imaging approach is therefore not radiation-free per se, but need not necessarily expose patients to extra radiation dose if CTs were taken for different clinical purpose anyway.

As a limitation of the approach it should be mentioned that morphological changes, occurring between CT examination and EIT measurement, will act as a wrong prior and may lead to corrupted reconstruction. Shape deformations during breathing are as well a known and often addressed problem in EIT, whose errors are currently accepted in all routinely applied reconstruction methods. Though a real thorax shape is unlikely to be more error prone than a static circle in reconstruction, the sensitivity of the approach to the mismatch between assumed lung shapes and actual form needs to be examined in future research. A further limitation of our study was a heuristical determination of the number of DCT coefficients. Additional examinations are necessary to define an optimal number of DCT coefficients, considering e.g. the inherently low resolution of EIT. Furthermore we only considered the lung shapes of a single slice; this has to be extended in future studies to investigate whether a 3D model of the lungs leads to better results. Additional studies with more patients are necessary to further evaluate the approach and to demonstrate the performance in combination with morphological data.

Although the images obtained with this method look quite attractive we want to point out that we have no evidence that the general resolution is improved, however it is proven that using the actual thorax contour for image reconstruction leads to better results^7^. Nevertheless we are confident that the approach described in this paper simplifies superposition of CT or MRI images with data obtained from EIT and therefore might help clinicians in image interpretation and diagnosis in a similar way as it is already done in the combination of PET or SPECT images and CT data.

## Conclusion

In conclusion, anomalies in ventilation distribution can be correlated with underlying morphological information, and vice versa the impact of pathological changes in lung tissue on ventilation distribution can be examined if the described approach is used for image reconstruction. We want to emphasize that EIT data might as well be used to generate functional images, e.g. regional time constant maps, which is only possible due to the high frame rate of EIT. Correlation of morphological images with EIT data might especially be useful for spontaneously breathing patients, suffering e.g. from asthma bronchiole, CF or COPD. For these patients we do not expect large changes in lung shape as it might happen in a ventilated patient e.g. during a recruitment maneuver. This means that for EIT the described method might be a step out of the intensive care unit, towards radiology and pulmonology, where merged CT-EIT images might be used to improve diagnostic accuracy and as a result patient outcome.

## Additional Information

**How to cite this article**: Schullcke, B. *et al*. Structural-functional lung imaging using a combined CT-EIT and a Discrete Cosine Transformation reconstruction method. *Sci. Rep.* **6**, 25951; doi: 10.1038/srep25951 (2016).

## Figures and Tables

**Table 1:** Patient overview.

Patient #	Disease type	Type of breathing	Used EIT device
1	COPD	Spontaneous tidal breathing	PulmoVista500
2	COPD, Lung emphysema	Spontaneous tidal breathing	PulmoVista500
3	Pneumonia	PEEP step (0–15 cmH2O)	Goe-MF II
4	Pneumonia	PEEP step (0–15 cmH2O)	Goe-MF II
5	COPD, Lung emphysema	Spontaneous tidal breathing	PulmoVista500

## References

[b1] Frerichs I. Electrical impedance tomography (eit) in applications related to lung and ventilation: a review of experimental and clinical activities. Physiological measurement 21, R1 (2000). 10847187 10.1088/0967-3334/21/2/201

[b2] Hinz J. . Regional ventilation by electrical impedance tomography: a comparison with ventilation scintigraphy in pigs. CHEST Journal 124, 314–322 (2003). 10.1378/chest.124.1.314 12853539

[b3] Zhao Z., Steinmann D., Frerichs I., Guttmann J. & Möller K. Research peep titration guided by ventilation homogeneity: a feasibility study using electrical impedance tomography. Critical care (London, England) 14, R8 (2010). 10.1186/cc8860 PMC2875520 20113520

[b4] Krueger-Ziolek S. . Positioning of electrode plane systematically influences eit imaging. Physiological measurement 36, 1109 (2015). 26006327 10.1088/0967-3334/36/6/1109

[b5] Leonhardt S. & Lachmann B. Electrical impedance tomography: the holy grail of ventilation and perfusion monitoring? Intensive care medicine 38, 1917–1929 (2012). 22992946 10.1007/s00134-012-2684-z

[b6] Brown B. Electrical impedance tomography (eit): a review. Journal of medical engineering & technology 27, 97–108 (2003). 12775455 10.1080/0309190021000059687

[b7] Grychtol B., Lionheart W. R., Bodenstein M., Wolf G. K. & Adler A. Impact of model shape mismatch on reconstruction quality in electrical impedance tomography. Medical Imaging, IEEE Transactions on 31, 1754–1760 (2012). 10.1109/TMI.2012.2200904 PMC7176467 22645263

[b8] Pikkemaat R., Tenbrock K., Lehmann S. & Leonhardt S. Electrical impedance tomography: New diagnostic possibilities using regional time constant maps. Appl Cardiopul P (ACP) 16, 212–225 (2012).

[b9] Zhao Z., Müller-Lisse U., Frerichs I., Fischer R. & Möller K. Regional airway obstruction in cystic fibrosis determined by electrical impedance tomography in comparison with high resolution ct. Physiological measurement 34, N107 (2013). 24150032 10.1088/0967-3334/34/11/N107

[b10] Vogt B. . Spatial and temporal heterogeneity of regional lung ventilation determined by electrical impedance tomography during pulmonary function testing. Journal of Applied Physiology 113, 1154–1161 (2012). 22898553 10.1152/japplphysiol.01630.2011

[b11] Milne S. & King G. G. Advanced imaging in copd: insights into pulmonary pathophysiology. Journal of thoracic disease 6, 1570 (2014). 25478198 10.3978/j.issn.2072-1439.2014.11.30 PMC4255158

[b12] Czernin J., Allen-Auerbach M. & Schelbert H. R. Improvements in cancer staging with pet/ct: literature-based evidence as of september 2006. Journal of Nuclear Medicine 48, 78S–88S (2007). 17204723

[b13] Kinahan P., Townsend D., Beyer T. & Sashin D. Attenuation correction for a combined 3d pet/ct scanner. Medical physics 25, 2046–2053 (1998). 9800714 10.1118/1.598392

[b14] Townsend D. W. Combined positron emission tomography-computed tomography: the historical perspective. In Seminars in Ultrasound, CT and MRI vol. 29, 232–235 (Elsevier, 2008). 10.1053/j.sult.2008.05.006 PMC2777694 18795489

[b15] Victorino J. A. . Imbalances in regional lung ventilation: a validation study on electrical impedance tomography. American Journal of Respiratory and Critical Care Medicine 169, 791–800 (2004). 14693669 10.1164/rccm.200301-133OC

[b16] Frerichs I. . Detection of local lung air content by electrical impedance tomography compared with electron beam ct. Journal of applied physiology 93, 660–666 (2002). 12133877 10.1152/japplphysiol.00081.2002

[b17] Kunst P., Noordegraaf A. V., Hoekstra O., Postmus P. & De Vries P. Ventilation and perfusion imaging by electrical impedance tomography: a comparison with radionuclide scanning. Physiological measurement 19, 481 (1998). 9863674 10.1088/0967-3334/19/4/003

[b18] Richard J. . Electrical impedance tomography compared to positron emission tomography for the measurement of regional lung ventilation: an experimental study. Critical Care 13, 1–9 (2009). 10.1186/cc7900 PMC2717448 19480694

[b19] Ferrario D. . Toward morphological thoracic eit: major signal sources correspond to respective organ locations in ct. Biomedical Engineering, IEEE Transactions on 59, 3000–3008 (2012). 10.1109/TBME.2012.2209116 22829362

[b20] Soleimani M. Computational aspects of low frequency electrical and electromagnetic tomography: A review study. Int. J. Numer. Anal. Model 5, 407–440 (2008).

[b21] Vauhkonen M., Kaipio J., Somersalo E. & Karjalainen P. Electrical impedance tomography with basis constraints. Inverse Problems 13, 523 (1997).

[b22] Hua P., Webster J. & Tompkins W. A regularised electrical impedance tomography reconstruction algorithm. Clinical Physics and Physiological Measurement 9, 137 (1988). 3240642 10.1088/0143-0815/9/4a/023

[b23] Winkler R. & Rieder A. Model-aware newton-type inversion scheme for electrical impedance tomography. Inverse Problems 31, 045009 (2015).

[b24] Soleimani M., Gómez-Laberge C. & Adler A. Imaging of conductivity changes and electrode movement in eit. Physiological measurement 27, S103 (2006). 16636402 10.1088/0967-3334/27/5/S09

[b25] Hartinger A. E., Guardo R., Adler A. & Gagnon H. Real-time management of faulty electrodes in electrical impedance tomography. Biomedical Engineering, IEEE Transactions on 56, 369–377 (2009). 10.1109/TBME.2008.2003103 19272943

[b26] Metherall P., Barber D., Smallwood R. & Brown B. Three dimensional electrical impedance tomography. Nature 380, 509–512 (1996). 8606768 10.1038/380509a0

[b27] Adler A., Dai T. & Lionheart W. R. Temporal image reconstruction in electrical impedance tomography. Physiological measurement 28, S1 (2007). 17664627 10.1088/0967-3334/28/7/S01

[b28] Vauhkonen M., Karjalainen P. A. & Kaipio J. P. A kalman filter approach to track fast impedance changes in electrical impedance tomography. Biomedical Engineering, IEEE Transactions on 45, 486–493 (1998). 10.1109/10.664204 9556965

[b29] Adler A. . Greit: a unified approach to 2d linear eit reconstruction of lung images. Physiological measurement 30, S35 (2009). 19491438 10.1088/0967-3334/30/6/S03

[b30] Antink C. H., Pikkemaat R., Malmivuo J. & Leonhardt S. A shape-based quality evaluation and reconstruction method for electrical impedance tomography. Physiological measurement 36, 1161 (2015). 26008150 10.1088/0967-3334/36/6/1161

[b31] Rahmati P., Soleimani M., Pulletz S., Frerichs I. & Adler A. Level-set-based reconstruction algorithm for eit lung images: first clinical results. Physiological measurement 33, 739 (2012). 22532379 10.1088/0967-3334/33/5/739

[b32] Borsic A., Graham B. M., Adler A. & Lionheart W. R. *In vivo* impedance imaging with total variation regularization. Medical Imaging, IEEE Transactions on 29, 44–54 (2010). 10.1109/TMI.2009.2022540 20051330

[b33] Blott B., Cox S., Daniell G., Caton M. & Nicole D. High fidelity imaging and high performance computing in nonlinear eit. Physiological measurement 21, 7 (2000). 10719994 10.1088/0967-3334/21/1/302

[b34] Dodd M. & Mueller J. L. A real-time d-bar algorithm for 2-d electrical impedance tomography data. Inverse problems and imaging (Springfield, Mo.) 8, 1013 (2014). 10.3934/ipi.2014.8.1013 PMC4414053 25937856

[b35] Harrach B. & Seo J. K. Exact shape-reconstruction by one-step linearization in electrical impedance tomography. SIAM Journal on Mathematical Analysis 42, 1505–1518 (2010).

[b36] Grychtol B. & Adler A. Uniform background assumption produces misleading lung eit images. Physiological measurement 34, 579 (2013). 23718942 10.1088/0967-3334/34/6/579

[b37] Vauhkonen M. *Electrical impedance tomography and prior information*. Ph.D. thesis, University of Kuopio, Finland (1997).

[b38] Graham B. M. *Enhancements in Electrical Impedance Tomography (EIT) image reconstruction for three-dimensional lung imaging.* Ph.D. thesis, University of Ottawa (Canada) (2007).

[b39] Polydorides N. & Lionheart W. R. A matlab toolkit for three-dimensional electrical impedance tomography: a contribution to the electrical impedance and diffuse optical reconstruction software project. Measurement Science and Technology 13, 1871 (2002).

[b40] Honerkamp J. & Weese J. Tikhonovs regularization method for ill-posed problems. Continuum Mechanics and Thermodynamics 2, 17–30 (1990).

[b41] Adler A. & Guardo R. Electrical impedance tomography: regularized imaging and contrast detection. Medical Imaging, IEEE Transactions on 15, 170–179 (1996). 10.1109/42.491418 18215899

[b42] Adler A. & Lionheart W. R. Uses and abuses of eidors: an extensible software base for eit. Physiological measurement 27, S25 (2006). 16636416 10.1088/0967-3334/27/5/S03

[b43] Schöberl J. Netgen an advancing front 2d/3d-mesh generator based on abstract rules. Computing and visualization in science 1, 41–52 (1997).

[b44] Adler A., Gaggero P. O. & Maimaitijiang Y. Adjacent stimulation and measurement patterns considered harmful. Physiological measurement 32, 731 (2011). 21646709 10.1088/0967-3334/32/7/S01

[b45] Wolf G. K. . Reversal of dependent lung collapse predicts response to lung recruitment in children with early acute lung injury. Pediatric Critical Care Medicine 13, 509–515 (2012). 22622650 10.1097/PCC.0b013e318245579c

[b46] Graham B. & Adler A. Objective selection of hyperparameter for eit. Physiological measurement 27, S65 (2006). 16636421 10.1088/0967-3334/27/5/S06

[b47] Lipponen A., Seppänen A. & Kaipio J. Electrical impedance tomography imaging with reduced-order model based on proper orthogonal decomposition. Journal of Electronic Imaging 22, 023008–023008 (2013).

[b48] Kaipio J. P., Kolehmainen V., Vauhkonen M. & Somersalo E. Inverse problems with structural prior information. Inverse problems 15, 713 (1999).

[b49] Borsic A., Lionheart W. R. & McLeod C. N. Generation of anisotropic-smoothness regularization filters for eit. Medical Imaging, IEEE Transactions on 21, 579–587 (2002). 10.1109/TMI.2002.800611 12166853

[b50] Woo E. J., Hua P., Webster J. G. & Tompkins W. J. Measuring lung resistivity using electrical impedance tomography. Biomedical Engineering, IEEE Transactions on 39, 756–760 (1992). 10.1109/10.142651 1516943

[b51] Glidewell M. & Ng K. T. Anatomically constrained electrical impedance tomography for anisotropic bodies via a two-step approach. Medical Imaging, IEEE Transactions on 14, 498–503 (1995). 10.1109/42.414615 18215854

[b52] Borsic A. *Regularisation methods for imaging from electrical measurements.* Ph.D. thesis, Oxford Brookes University (2002).

[b53] Vauhkonen M., Vadasz D., Karjalainen P. A., Somersalo E. & Kaipio J. P. Tikhonov regularization and prior information in electrical impedance tomography. Medical Imaging, IEEE Transactions on 17, 285–293 (1998). 10.1109/42.700740 9688160

[b54] Krishnan K., Liu J. & Kohli K. Feature-space assessment of electrical impedance tomography coregistered with computed tomography in detecting multiple contrast targets. Medical physics 41, 061903 (2014). 24877814 10.1118/1.4873326

